# Terlipressin for septic shock patients: a meta-analysis of randomized controlled study

**DOI:** 10.1186/s40560-019-0369-1

**Published:** 2019-03-12

**Authors:** Yibing Zhu, Huibin Huang, Xiuming Xi, Bin Du

**Affiliations:** 1 ICU, Fuxing Hospital, Capital Medical University, Peking, China; 2 Medical ICU, Peking Union Medical College Hospital, Peking Union Medical College and Chinese Academy of Medical Sciences, Peking, China; 3 Department of Critical Care Medicine, the First Affiliated Hospital of Fujian Medical University, Fuzhou, China

**Keywords:** Terlipressin, Catecholamines, Septic shock, Intensive care unit, Meta-analysis

## Abstract

**Background:**

Catecholamines are commonly used in septic shock but face limitations of their hypo-responsiveness and adverse events due to high dose. Terlipressin is a synthetic vasopressin analog with greater selectivity for the V1-receptor. A meta-analysis was conducted to evaluate the efficacy and safety of terlipressin in septic shock.

**Methods:**

We searched for relevant studies in PubMed, Embase, and the Cochrane database from inception up to July 15, 2018. Randomized controlled trials (RCTs) were included if they reported data on any of the predefined outcomes in patients with septic shock and managed with terlipressin or any catecholamines. Results were expressed as risk ratio (RR) or mean difference (MD) with accompanying 95% confidence interval (CI). Heterogeneity, subgroup analysis, sensitivity analysis, and publication bias were explored.

**Results:**

Ten studies with 928 patients were included. Despite the shorter duration of mechanical ventilation, use of terlipressin did not reduce the risk of mortality (RR = 0.94; 95% CI, 0.85 to 1.05; *I*^2^ = 0%; *P* = 0.28) when compared with control. This finding was confirmed by further subgroup and sensitivity analyses. In addition, lactate clearance, length of stay in ICU or hospital, total adverse events, digital ischemia, and arrhythmia were also similar between groups, while terlipressin was associated with shorter duration of mechanical ventilation and less norepinephrine requirements.

**Conclusions:**

Current results suggest terlipressin did not show added survival benefit in septic shock therapy when compared with catecholamines.

**Electronic supplementary material:**

The online version of this article (10.1186/s40560-019-0369-1) contains supplementary material, which is available to authorized users.

## Key messages

- Terlipressin did not show added survival benefit in septic shock therapy when compared with catecholamines.
- Lactate clearance, length of stay in ICU or hospital, total adverse events, digital ischemia, and arrhythmia were similar between groups.
- Terlipressin seemed to be associated with decreased duration of MV and norepinephrine requirement.

## Introduction

Septic shock is a major life-threatening and refractory vasodilatory shock in the intensive care unit (ICU). Vasopressor therapy is crucial in the management of septic shock to achieve target arterial blood pressure. Catecholamine has long been first and foremost recommended vasopressors [1]. However, some patients may remain refractory to this agent, which is also known as catecholamine-resistant septic shock [2, 3]. Moreover, high-dose catecholamine therapy may lead to potential side effects such as increased myocardial oxygen consumption, lethal arrhythmias, and even the high risk of mortality [4]. Thus, the selection of other vasoactive drugs as alternative or accessory that may benefit septic shock while avoiding unnecessary side effects is important.

Vasopressin is an endogenously released peptide hormone and exerts vasoconstriction effect via stimulating specific receptors mainly V1 receptors [5–7]. A previous study indicated the survival benefit in terlipressin [8]. Recent guideline also referred the use of vasopressin as potential rescue agents in catecholamine-refractory septic shock [1]. However, in the vasopressin and septic shock trial (VASST), low-dose AVP failed to reduce overall mortality compared with norepinephrine in patients with septic shock [6]. One of potential explanation for this is that vasopressin has no selectivity for V1 receptors and may also activate other receptors, thus leading to a variety of adverse effects, such as decreased cardiac output, thrombocytopenia, hyponatremia, or hyperbilirubinemia [5–7]. Therefore, terlipressin (tricyl-lysine vasopressin), a synthetic analog of vasopressin, has attracted attention for its similar pharmacodynamic profile but greater selectivity of V1 receptor [5–7].

In some preliminary studies, terlipressin administration seems beneficial to less norepinephrine requirement, improved hemodynamic status and more creatinine clearance during septic shock therapy [8–10]. However, two meta-analyses evaluating vasopressin/terlipressin therapy for vasodilatory shock looked into a subgroup of terlipressin [11, 12], suggesting that terlipressin did not improve survival in septic shock. These findings were limited for basing on an evaluation of around 100 patients from three unblinded randomized controlled trials (RCTs) published between the year 2005 and 2009 [9, 13, 14], and some of important outcomes such as effect on the dose of catecholamine, duration of mechanical ventilation (MV), and length of stay (LOS) in ICU or hospital were not considered in the two previous meta-analyses [11, 12]. Mårtensson and Gordon [15] conducted a meta-analysis in their commentary on a newly published RCT [8]; however, they included only four trials without systematically review.

Recently, several studies have evaluated the effect of terlipressin for patients with septic shock. Therefore, with the aid of the increased power of meta-analytic techniques, we aimed to review the relevant and available published RCTs to test the hypothesis that, compared with conventional vasopressors, terlipressin may decrease overall mortality in patients with septic shock.

## Materials and methods

### Search strategy and selection criteria

The study protocol was published [16] and registered in the PROSPERO international prospective register of systematic reviews (registration number CRD42018104924). We searched RCTs in PubMed, Embase, and Cochrane databases from inception through July 15, 2018, to identify potentially relevant studies. A search strategy was developed for PubMed and the other databases (Additional file 1: Search strategy). Our research was limited to RCTs with no language restriction. Reference lists of relative articles were also reviewed.

Studies were included if they met the following criteria: (1) RCTs; (2) ICU patients with septic shock; (3) intervention: patients receiving terlipressin, regardless of dosage, frequency, duration, and administration routes; any open-label catecholamines can be added whenever needed; (4) control: patients receiving any catecholamines; and (5) reporting any of the following outcomes: mortality, ICU LOS, duration of MV, catecholamines requirement, lactate clearance, and adverse events (AEs). Detailed statements of patient, intervention, comparison, and definitions are presented in Additional file 2: Table S3. Studies were excluded if they enrolled pregnant or breastfeeding woman or if they were only in abstract form, meeting reports. The studies were also excluded if their data were missing or incomplete or the study authors were unreachable or did not reply if additional information from their trials was required.

### Data extraction and outcomes

Data extraction was undertaken by two authors (HBH and YBZ) independently from included RCTs on the first author, year of publication, study design, sample size, setting, treatment algorithms of terlipressin and control groups, prognostic index, and methodological quality, as well as all outcomes of interest. The primary outcome was that all causes mortality at the longest follow-up available. Secondary outcomes included length of stay in ICU and hospital, duration of MV, lactate clearance rate in 24 h, catecholamines requirement, and AEs. Discordant opinions between the two reviewers (HBH and YBZ) were discussed until consensus was reached. If consensus could not be reached, a consulting group including two experts (XMX and BD) resolved the disagreements.

### Quality assessment

The quality of studies was evaluated using the risk of bias tool recommended by the Cochrane Collaboration [17]. We assigned a value of high, unclear, or low to the following items: sequence generation, allocation concealment, blinding, incomplete outcome data, selective outcome reporting, and other sources of bias. Any discrepancies were identified and resolved through discussion. “The quality of evidence resulting from this systematic review was evaluated using the GRADE (Grades of Recommendation Assessment, Development and Evaluation) methodology” [18, 19].

### Statistical analysis

The results from all relevant studies were combined to estimate the pooled risk ratio (RR) and associated 95% confidence intervals (CIs) for dichotomous outcomes. As to the continuous outcomes, mean differences (MD) and 95% CI were estimated as the effect results. Some studies reported median as the measure of treatment effect, with accompanying interquartile range (IQR). Before data analysis, we estimated mean from median and standard deviations (SD) from IQR using the methods described in previous studies [20]. Heterogeneity was tested by using the *I*^2^ statistic. Inverse variance random-effects models were applied for the data analysis. Testing the robustness of our primary outcome and exploring the potential influence factors, we conducted subgroup analyses by pooled studies with the following: (1) type of catecholamine as control (norepinephrine or other catecholamines), (2) administration of terlipressin (bolus or continuous), (3) terlipressin dose (> 4 mg/d; 2–4 mg/d or < 2 mg/d), (4) study design (blinded or unblinded), and (5) published year (before year 2010 or after year 2010). We also conducted sensitivity analyses on mortality by pooling studies only focusing on: (1) 28-day mortality, (2) ICU mortality, (3) hospital mortality, (4) more severe septic shock (defined as catecholamine-resistant septic shock or patients received more than 15 μg/min norepinephrine at randomization), (5) less severe septic shock, (6) studies of exclusion of the largest trial, and (7) studies of exclusion of pediatrics. Publication bias was evaluated by visually inspecting funnel plots when at least 10 studies were included in this meta-analysis. All analyses were performed using Review Manager, version 5.3.

## Results

### Trial selection and characteristics

**Fig. 1:**
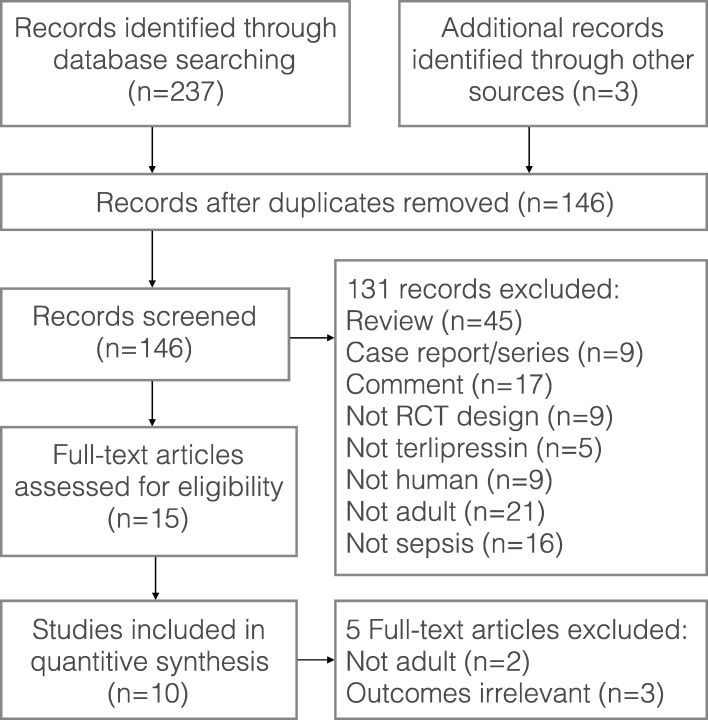
Flow diagram of the process for identification of the included studies

The literature search yielded 146 records through database searching, and 10 RCTs fulfilling the inclusion criteria were eligible for final analysis. The flow chart of our search strategy is presented in Fig. 1. The main characteristics of included studies are shown in Table 1, while the Cochrane risk of bias score that varied across these studies was summarized in Additional file 3: Figure S1. The included studies were conducted in six medical-surgical ICUs, one pediatric ICU, one liver ICU, and one trauma ICU. Nine out of the 10 RCTs were single-center studies. A total of 948 patients were included in the final analysis (sample size ranging from 20 to 526 patients), with 471 patients in terlipressin group and 477 patients in control group. Nine trials included adults and one trial included children. As to the catecholamines used as control, norepinephrine and dopamine were used in eight studies and one study, respectively, while both dopamine and dobutamine were used in another study. Terlipressin dose and duration varied among the included trials. An initial target mean arterial pressure of 65–75 mmHg was recommended by all the included RCTs.

**Table 1:** Characteristics of included studies

Study ID	Setting	Type of shock		Patient characteristics					
			Number of participants	Mean age (years)	Prognostic index	Baseline mean MAP (mmHg)	Baseline mean LAC (mmol/L)	Regimen	Target MAP (mmHg)
Albanèse 2005 [13]	Mix-ICU	SS	TP (*n* = 10)	66	28 (A II)	54	NR	TP 1 mg bolus once or twice	65–75
			NE (*n* = 10)	65	29 (A II)	54		NE 0.3 μg/kg/min followed by 0.3 μg/kg/min increments at 4-min intervals	
Yildizdas 2008 [14]	PICU	CRSS	TP (*n* = 30)	2	26.4 (P)	56	NR	TP 20 μg/kg bolus every 6 h for at most 96 h	> 2 SD for age
			DA+DB (*n* = 28)	2	27.9 (P)	56		DA 0–20 μg/kg/min, DB 0-15 μg/kg/min, if need E 0–2 μg/kg/min	
Morelli 2008 [9]	Mix-ICU	SS	TP+NE (*n* = 19)	66	60 (S II)	74	3	TP 1 mg bolus, NE 0.9 μg/kg/min	65–75
			TP+DB+NE (*n* = 20)	66	61 (S II)	72	3	TP 1 mg bolus, DB 3–20 μg/kg/min, NE 0.9 μg/kg/min	
			NE (*n* = 20)	67	59 (S II)	73	3	NE titrated	
Morelli 2009 [10]	Mix-ICU	SS	TP (*n* = 15)	67	62 (S II)	53	3	TP 1.3 μg/kg/h	65–75
			NE (*n* = 15)	64	58 (S II)	54	3	NE 15 μg/min	
Svoboda 2012 [2]	TICU	CRSS	TP (*n* = 13)	70	18 (SOFA)	71	7	TP 4 mg/24 h	65–75
			NE (*n* = 17)	75	18 (SOFA)	74	8	NE titrated	
Hua 2013 [21]	Mix-ICU	ARDS+shock	TP (*n* = 16)	57	19 (A II); 42 (S II)	59	NR	TP 1.4 μg/kg/h	65–75
			DA (*n* = 16)	52	18 (A II); 48 (S II)	58		DA 0–20 μg/kg/min	
Xiao 2015 [22]	Mix-ICU	SS	TP+NE (*n* = 15)	62	NR	66	3.2	TP 1.3 μg/kg/h	65–90
			NE (*n* = 17)	63		64	3.6	NE titrated	
Choudhury 2016 [24]	LICU	Cirrhosis+SS	TP (*n* = 42)	47	14 (SOFA)	61	3	TP 1.3–5.2 μg/min	≥ 65
			NE (n = 42)	48	15 (SOFA)	60	3	NE 7.5-60 μg/min	
Chen 2017 [23]	Mix-ICU	ARDS+SS	TP (*n* = 31)	59	23 (A II)	55	NR	TP 0.01–0.04 U/min	65–75
			NE (*n* = 26)	56	21 (A II)	54		NE titrated	
Liu 2018 [8]	Mix-ICU	SS	TP (*N* = 260)	61	19 (A II); 11 (SOFA)	68	4	TP 20-160 μg/h	65–75
			NE (*n* = 266)	61	19 (A II); 11 (SOFA)	68	4	NE 4-160 μg/h	

*A II* acute physiology and chronic health evaluation II, *ARDS* acute respiratory distress syndrome, *CRSS* catecholamine-resistant septic shock, *DA* dopamine, *DB* dobutamine, *E* epinephrine, *LAC* lactate, *LICU* liver intensive care unit, *MAP* mean arterial pressure, *Mix-ICU* intensive care unit, *NE* noradrenaline, *NR* not report, *P* pediatric risk of mortality, *PICU* pediatric intensive care unit, *S II* simplified acute physiologic score II, *SD* standard deviation, *SOFA* sequential organ failure assessment score, *SS* septic shock, *TICU* trauma intensive care unit, *TP* terlipressin

### Primary outcome

**Fig. 2:**
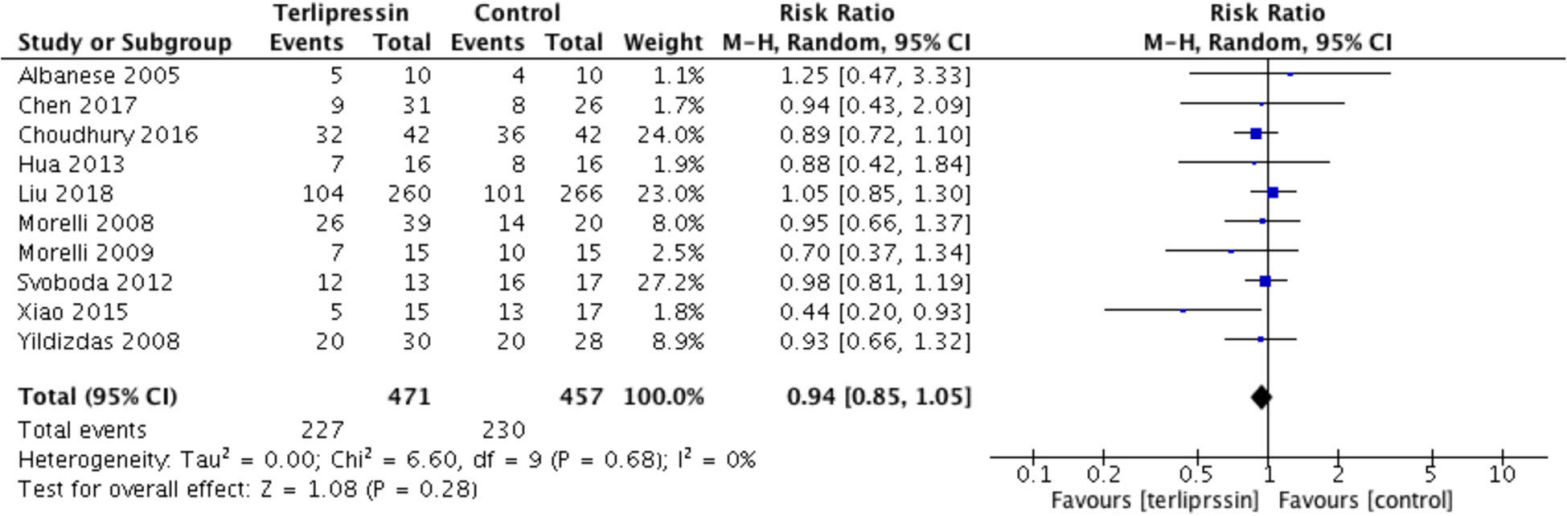
Forest plot. No significant difference in mortality

All the 10 included RCTs (*n* = 928 patients) presented results for the overall mortality. The pooled analysis showed that, compared with the control group, the terlipressin group did not result in a significant change in risk of mortality (RR = 0.94; 95% CI, 0.85 to 1.05; *I*^2^ = 0%; *P* = 0.28) (Fig. 2). Although no significant heterogeneity was shown, we proceeded to perform subgroup analyses across predefined important clinical factors. In general, all the subgroup analyses confirmed similar mortality rate among groups. Sensitivity analyses were subsequently conducted, suggesting that when only 28-day mortality, ICU mortality, hospital mortality, more severe septic shock, studies of exclusion of the largest trial, or studies of exclusion of pediatrics were considered, there was no difference between groups. Details of the results of subgroup analyses and sensitivity analyses are shown in Table 2. Using overall mortality as an outcome, the funnel plot suggested the presence of publication bias. (Additional file 4: Figure S2).

**Table 2:** Further subgroup analysis and sensitivity analyses on primary outcome of mortality rate

	Groups	References	Patient number (TP/Ctrl)	Event (TP/Ctrl)	RR (95%CI)	*I* ^*2*^	*P*
Subgroup analyses							
Type of control	NE	[2, 8–10, 13, 21–23]	425/413	200/202	0.95 (0.85,1.06)	0%	0.33
	DA	[14, 20]	46/44	27/28	0.92 (0.67,1.26)	0%	0.61
Published year	After 2010	[2, 5, 20–23]	377/384	169/182	0.95 (0.84,1.07)	8%	0.40
	Before 2010	[9, 10, 13, 14]	94/73	58/48	0.92 (0.74,1.16)	0%	0.49
Study designed	Blinded	[8, 21]	275/283	109/114	0.73 (0.31,1.72)	79%	0.48
	Unblinded	[2, 9, 10, 13, 14, 20, 22, 23]	196/174	118/116	0.93 (0.83,1.05)	0%	0.24
TP dose	< 2 mg/d	[9, 10, 13, 22]	95/71	47/36	0.92 (0.69,1.22)	0%	0.55
	2–4 mg/d	[2, 8, 14, 20, 21]	334/344	148/158	0.96 (0.82,1.12)	0%	0.61
	> 4 mg/d	[23]	42/42	32/36	0.89 (0.72,1.10)		0.27
Administration route	Bolus	[9, 13, 21]	64/47	36/31	0.81 (0.46,1.40)	51%	0.44
	Continuous infusion	[2, 8, 10, 14, 20, 22, 23]	407/410	191/199	0.96 (0.86,1.07)	0%	0.42
Sensitivity analyses							
	28-day mortality	[2, 20, 22, 23]	102/101	60/68	0.93 (0.81,1.07)	0%	0.34
	ICU mortality	[9, 10, 14]	84/63	53/44	0.91 (0.72,1.14)	0%	0.41
	Hospital mortality	[13]	10/10	5/4	1.25 (0.4,3.33)		0.66
	90-day mortality	[2]	13/17	12/16	0.98 (0.81,1.19)		0.85
	More severe septic shock	[2, 9, 10, 13, 14]	107/90	70/64	0.96 (0.82,1.11)	0%	0.55
	Less severe septic shock	[8, 20–23]	364/367	157/166	0.72 (0.40,1.29)	43%	0.14
	Exclusion of the largest trial	[2, 9, 10, 13, 14, 20–23]	211/191	123/129	0.91 (0.81,1.03)	0%	0.14
	Exclusion of pediatric patients	[2, 8–10, 13, 20–23]	441/429	207/210	0.95 (0.85,1.05)	0%	0.31

*Ctrl* control group, *DA* dopamine, *ICU* intensive care unit, *NE* norepinephrine, *RR* risk ratio, *TP* terlipressin group

### Secondary outcomes

**Fig. 3:**
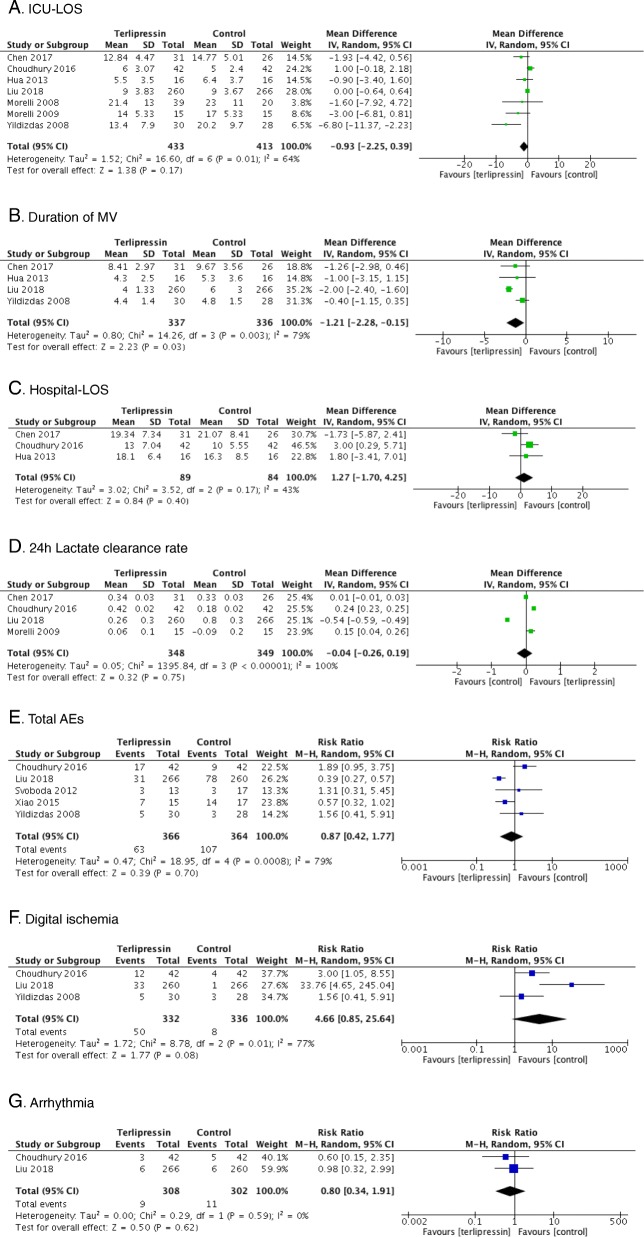
Forest plots. Secondary outcomes of **a** ICU-LOS, **b** duration of MV, **c** hospital-LOS, **d** 24 h lactate clearance rate, **e** total AEs, **f** digital ischemia, **g** arrhythmia. ICU intensive care unit, LOS length of stay, MV mechanical ventilation, AEs adverse events

Seven trials [8, 9, 14, 21–24] reported ICU-LOS as an outcome, which was similar between the terlipressin and control groups (*n* = 846; MD = − 0.93 days; 95% CI, − 2.25 to 0.39; *I*^2^ = 64%; *P* = 0.17) (Fig. 3a). Data from four studies [8, 14, 21, 23] found that use of terlipressin was associated with a shorter duration of MV (*n* = 675; MD = − 1.21 days; 95% CI, − 2.28 to − 0.15; *I*^2^ = 79%; *P* = 0.03) (Fig. 3b). Hospital-LOS was available in three studies [21, 23, 24], which was also similar between groups (*n* = 173; MD = 1.27; 95% CI, − 1.70 to 4.25; *I*^2^ = 43%; *P* = 0.40) (Fig. 3c). Data of lactate clearance rate in 24 h was extracted in four studies [8, 22–24], and no significant difference was found (4 trials, *n* = 697, RR = − 0.04; 95% CI, − 0.26 to 0.19; I2 = 100%; *P* = 0.75) (Fig. 3d). Three studies [8, 9, 22] focused on outcome of norepinephrine requirement, indicating less norepinephrine using in terlipressin group (3 trials, *n* = 590, MD = − 0.18; 95% CI, − 0.20 to − 0.17; *I*^2^ = 99%, *P* < 0.00001). Five RCTs [2, 8, 14, 22, 24] presented data regarding total AEs (Additional file 5: Table S1). The pooled data found that total AEs was similar between groups. There was no significant difference in total AEs (5 trials, *n* = 730, RR = 0.87; 95% CI, 0.42 to 1.77; *I*^2^ = 79%; *P* = 0.70). (Fig. 3e). Arrhythmia [8, 24] and digital ischemia [8, 14, 24] were the adverse events that were reported by more than two trials. When pooled, no difference was found between the two groups in outcome of arrhythmia (2 trials, *n* = 610, RR = 0.80; 95% CI, 0.34 to 1.91; *I*^2^ = 0%; *P* = 0.62) (Fig. 3g), whereas a tendency showed that digital ischemia was more common in the terlipressin group (3 trials, *n* = 668, RR = 4.66; 95% CI, 0.85 to 25.64; *I*^2^ = 77%; *P* = 0.08). (Fig. 3f). A summary of results and quality of evidence for each outcome pooled is shown in Additional file 6: Table S2.

## Discussion

In the current meta-analysis, we included only RCTs and compared the use of terlipressin with catecholamines in patients with septic shock. Terlipressin administration failed to decrease overall mortality, and this finding was confirmed by further analyses. Lactate clearance, ICU or hospital LOS, and total AEs were comparable between groups. In addition, use of terlipressin seemed to be associated with less norepinephrine requirement, shorter duration of MV, and near significant increased risk of digital ischemia.

Our findings expanded on the earlier meta-analyses to provide a more sufficient evidence for use of terlipressin in patients with septic shock [11, 12]. First, we expanded the previous meta-analysis by including additional seven RCTs published between 2012 and 2018 [2, 8, 13, 14, 22, 23], with more power to assess this effect. Second, our main finding was confirmed by further subgroups and sensitivity analyses based on various clinical characteristics. Finally, we also evaluated other related important outcomes, such as norepinephrine requirement, lactate clearance, hospital and ICU-LOS, and found no differences between groups, thus providing evidence of the robustness of our main finding.

Detailed explanation of the negative results of terlipressin is beyond the scope of a meta-analysis, but several topics related to the drug that may affect our main outcome merit further discussion. First, different terlipressin dose regimens were adopted by the included trials. This is because no equivalent dose of terlipressin compared to any catecholamines has been reported. Previous studies showed that terlipressin may be safe with low-dose infusion (about 110μg/h) [10], and the overall AEs increased when higher dosage and longer duration were required [15, 25]. However, subgroups of different terlipressin dose regimens showed an effect on the mortality when compared terlipressin with control group.

Second, administration route of terlipressin varied among the included studies, with some studies evaluating intermittent bolus doses [9, 13, 14], while some studies evaluating continuous infusion of a fixed dose [10, 21, 22] or titrating [8, 23, 24] to achieve target arterial blood pressure. In the preliminary studies, terlipressin was usually given as a single bolus [9, 13], mainly referred to the dosage used in gastroenterological practice [10, 24, 26]. However, bolus administration of terlipressin has been associated with several serious AEs such as myocardial ischemia, reduced cardiac output, and sudden or strong rebound effects [2, 13, 21, 27, 28]. Therefore, continuous infusion of terlipressin was selected by most of the recent studies to reduce these AEs. Interestingly, none of the included studies in current meta-analysis using terlipressin bolus regimen reported the above AEs, while two included studies using terlipressin-infusing regimen suggested no differences in myocardial ischemia between groups [2, 8]. Furthermore, our subanalyses on types of administration routes of terlipressin (intermittent bolus vs. continuous infusion form) also showed no differences in mortality between terlipressin and control groups.

Third, the included studies enrolling patients differed in severity of septic shock. It is usually considered that late-phase and catecholamine-refractory shock may be inclined to more severe septic shock, thus leading to a greater risk of mortality [2, 9, 10, 13, 14]. Vasopressin and terlipressin are recommended in patients’ refractory to other conventional vasopressor therapies. Hence, we further analyzed subgroups in varied severity of septic shock. Once again, no effect on mortality rate was found between terlipressin and control groups when only the studies of more severe septic shock were considered. One possible explanation for the insufficient effect of terlipressin is that administration started too late, when the septic shock became irreversible.

In words, though terlipressin is expected to be an alternative to vasopressin for its higher selectivity for V1-receptors and a longer effective half-life [5–7], it failed to improve overall mortality in patients with septic shock based on the current results. We consider terlipressin, as well as vasopressin, of multiple intervention for septic shock patients, rather than alternatives for catecholamines. In fact, from the point of view of pharmacology, the pharmacokinetics of tricyl-lysine vasopressin, the active metabolite of terlipressin, has not been established during septic shock therapy. On the other hand, to date, there has been no robust evidence of survival benefit from terlipressin in comparison with vasopressin in patients with septic shock [10–12].

So far, the pathophysiology of septic shock in ICU patients remains poorly understood. The therapies of septic shock may be multiple, including adequate fluid resuscitation, early application of antibiotic, or multiple organ support therapy [1]. Therefore, individualized regimen of septic shock management based on identification of mechanisms involved may be needed.

As to AEs, our results showed that the terlipressin and control groups had the same rate of total AEs. Further analysis showed that terlipressin was associated with numerically higher risk of digital ischemia than control treatment. Inadequately fluid-resuscitated, terlipressin-induced decreased cardiac index and high-dose terlipressin may contribute to such adverse ischemic events [2, 22, 24]. However, only five of the included studies [2, 8, 14, 22, 24] reported the total AEs and three [2, 22, 24] provided data on all kinds of AEs for analysis, which may not fully reflect the AEs from terlipressin during the study period.

As the most advanced evidence so far, the current meta-analysis showed no inferiority of terlipressin in comparison with catecholamines. It implies that terlipressin could be of great value for septic shock patients. Additional research are needed to further explore the optimum administration pattern and dose.

Our meta-analysis has several limitations. First, most of the included RCTs had a sample size of fewer than 100 patients, which might be subject to overestimation of effect size [29]. Small-study effects might lead to publication bias [29]. Second, significant heterogeneity was observed in some of our outcomes. Differences among included trials with regard to the adopted terlipressin-dosing regimen, types of open-label catecholamines used, timing and duration of terlipressin or control drugs, and other conventional therapies during septic shock might lead to the observed heterogeneity and further impair the robustness of our findings. Third, causes of septic shock and underlying diseases vary across included studies. The original plan of subgroup analysis to further explore studies based on the above diversities was hampered by insufficient data. Fourth, although predefined subgroup analyses had been performed, the results should be interpreted with caution due to the small number of patients in some outcomes. In addition, most of the included RCTs (8 of 10) were unblinded, which leads to suboptimal quality and inevitable risks of bias to a large extent.

## Conclusions

Current results suggest terlipressin did not show added survival benefit in septic shock therapy when compared with catecholamines. There was no significant difference in ICU-LOS, hospital-LOS, total AEs, digital ischemia, and lactate clearance. Terlipressin seemed to be associated with decreased duration of mechanical ventilation and norepinephrine requirements.

## Additional files

Additional file 1: Search strategy. (PDF 20 kb)

Additional file 2: **Table S3.** Detailed statements of definitions. (PDF 252 kb)

Additional file 3: **Figure S1.** Risk of bias. (PDF 3192 kb)

Additional file 4: **Figure S2.** Funnel plots. Funnel plots were generally asymmetrical. The hollow dots and dotted line indicate individual studies and 95% confidence intervals, respectively. (PDF 27 kb)

Additional file 5: **Table S1.** Adverse events reported by included studies. (XLSX 489 kb)

Additional file 6: **Table S2.** Summary of outcomes for the effect of Terlipressin in septic shock patients. (DOCX 21 kb)

## Abbreviations

AEs: Adverse events
AVP: Arginine vasopressin
CI: Confidence interval
ICU: Intensive care unit
IQR: Interquartile range
LOS: Length of stay
MD: Mean difference
RCTs: Randomized controlled trials
RR: Risk ratio
S II: Simplified acute physiology score II
SD: Standard deviations
