# Supplementary material for: Terlipressin for septic shock patients: a meta-analysis of randomized controlled study
Source: J Intensive Care. 2019 Mar 12;7:16. doi: 10.1186/s40560-019-0369-1 (PMC6419496; doi:10.1186/s40560-019-0369-1)
Supplement: Supplementary file 1 — Search strategy. (PDF 20 kb) [file 40560_2019_369_MOESM1_ESM.pdf]

Search Strategy

Search included: Cochrane Stroke Trials Registry to 15, July 2018 PubMed to 15, July 2018 Embase to 15, July 2018

PubMed:

1. terlipressin [ALL]
2. terlipressin [Mesh Terms]
3. vasopressin analogue [ALL]
4. vasopressin analog [ALL]
5. or/1-4
6. sepsis [ALL]
7. sepsis [Mesh Terms]
8. septic shock [ALL]
9. septic shock [Mesh Terms]
10. or/6-9
11. 5 and 10

148 hits on 15, July 2018

Embase

1. terlipressin [ALL]
2. vasopressin analogue [ALL] 3. vasopressin analog [ALL]
4. or/1-3
5. sepsis [ALL]
6. septic shock [ALL]
7. or/5-6
8. 4 and 7

30 hits on 15, July 2018

Cochrane library

1. terlipressin [ALL]
2. vasopressin analogue [ALL] 3. vasopressin analog [ALL]
4. or/1-3
5. sepsis [ALL]
6. septic shock [ALL]
7. or/5-6
8. 4 and 7

59 hits on 15, July 2018
