# Supplementary material for: Terlipressin for septic shock patients: a meta-analysis of randomized controlled study
Source: J Intensive Care. 2019 Mar 12;7:16. doi: 10.1186/s40560-019-0369-1 (PMC6419496; doi:10.1186/s40560-019-0369-1)
Supplement: Supplementary file 2 — Table S3. Detailed statements of definitions. (PDF 252 kb) [file 40560_2019_369_MOESM2_ESM.pdf]

Table S3 Detailed statements of definitions

Objective	Definition
Patient	ICU patients with septic shock were subjected to this study.
Age	We included patients of all age.
Septic shock	Patients developed shock due to sepsis; we considered the patients in development of septic shock if the authors of RCTs reported so.
ICU	We included all types of ICU, including MICU, SICU, PICU, Mix-ICU, LICU or any other kind.
Intervention	Terlipressin for vasopressor therapy in septic shock patients.
Terlipressin	Terlipressin regardless of dosage, frequency, duration and administration routes were included.
Comparison	Catecholamines for vasopressor therapy in septic shock patients.
Catecholamines	Catecholamines including norepinephrine, dopamine, dobutamine or any other kind.
Outcome	The primary outcome was mortality; secondary outcomes including ICU LOS, duration of MV, lactate clearance, catecholamines requirement, and adverse events.
Mortality	We reported all causes mortality at the longest follow up available; mortality measured in days.
ICU LOS	ICU LOS was from ICU admission to discharge; ICU LOS measured in days.
Duration of MV	Duration of MV was from initiation of MV to weaning; duration of MV measured in days.
Catecholamines requirement	Catecholamines requirement referred to open-label catecholamines administrated in experimental and/or control group, if the authors of RCTs reported any; Catecholamines requirement measured in µg/kg/min.
Lactate clearance	Lactate clearance referred to lactate clearance rate in 12 and/or 24 hours, if the authors of RCTs reported any; lactate clearance measured in percentage.

ICU intensive care unit, LICU liver intensive care unit, LOS length of stay, MICU medical intensive care unit, Mix-ICU intensive care unit, PICU pediatric intensive care unit, RCT randomized controlled study SICU surgical intensive care unit
