# Supplementary material for: Terlipressin for septic shock patients: a meta-analysis of randomized controlled study
Source: J Intensive Care. 2019 Mar 12;7:16. doi: 10.1186/s40560-019-0369-1 (PMC6419496; doi:10.1186/s40560-019-0369-1)
Supplement: Supplementary file 3 — Figure S1. Risk of bias. (PDF 3192 kb) [file 40560_2019_369_MOESM3_ESM.pdf]

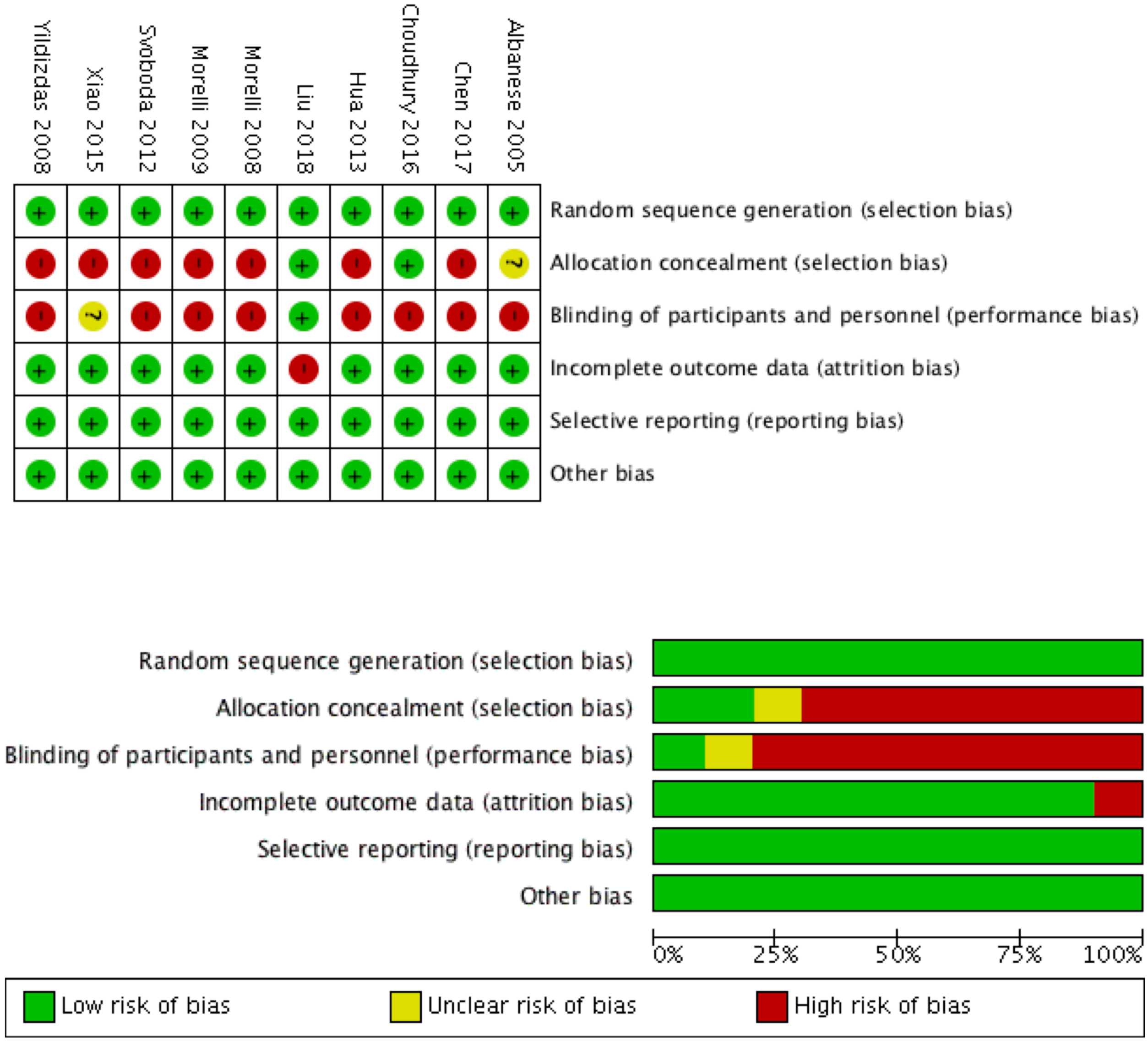

Fig. S1 Risk of bias.
