# Supplementary material for: Terlipressin for septic shock patients: a meta-analysis of randomized controlled study
Source: J Intensive Care. 2019 Mar 12;7:16. doi: 10.1186/s40560-019-0369-1 (PMC6419496; doi:10.1186/s40560-019-0369-1)
Supplement: Supplementary file 4 — Figure S2. Funnel plots. Funnel plots were generally asymmetrical. The hollow dots and dotted line indicate individual studies and 95% confidence intervals, respectively. (PDF 27 kb) [file 40560_2019_369_MOESM4_ESM.pdf]

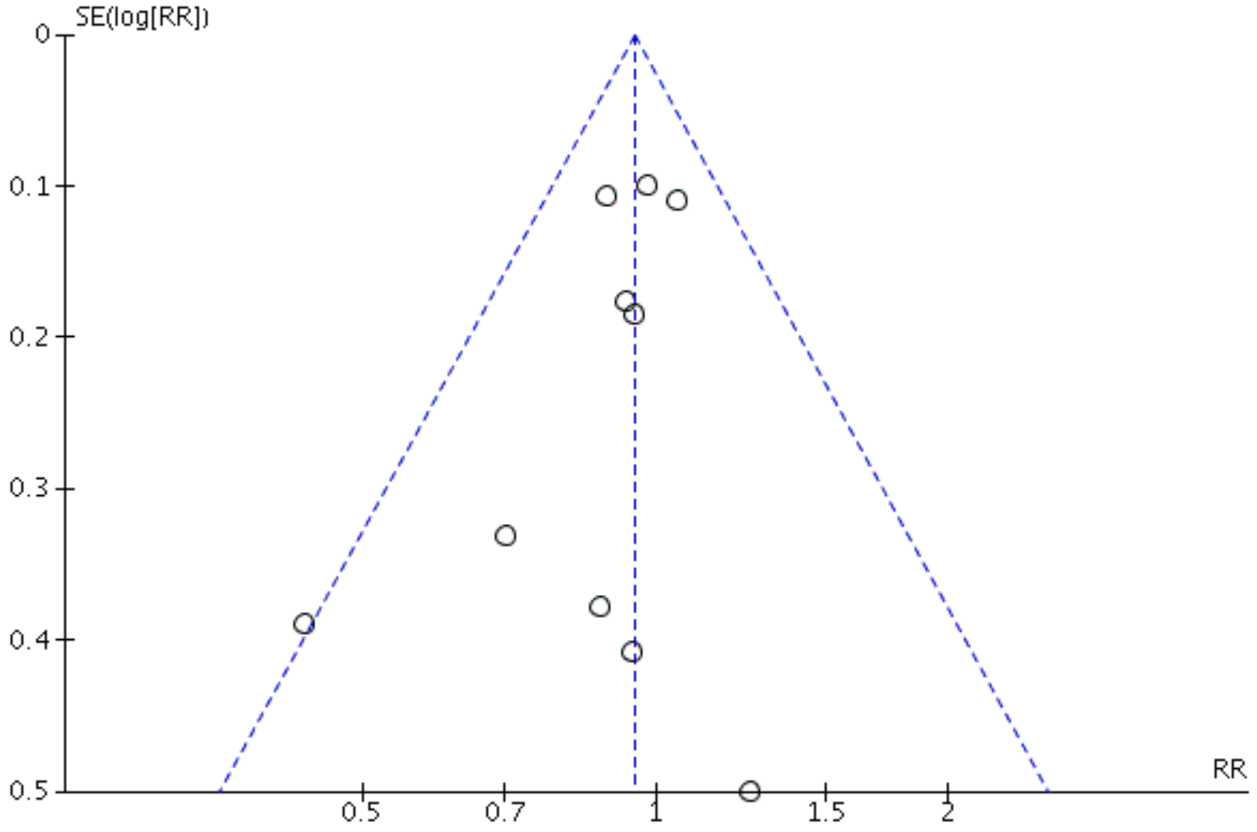

Fig. S2 Funnel plots. Funnel plots were generally asymmetrical. The hollow dots and dotted line indicate individual studies and 95% confidence intervals, respectively.
