# Supplementary material for: Terlipressin for septic shock patients: a meta-analysis of randomized controlled study
Source: J Intensive Care. 2019 Mar 12;7:16. doi: 10.1186/s40560-019-0369-1 (PMC6419496; doi:10.1186/s40560-019-0369-1)
Supplement: Supplementary file 6 — Table S2. Summary of outcomes for the effect of Terlipressin in septic shock patients. (DOCX 21 kb) [file 40560_2019_369_MOESM6_ESM.docx]

**Table S2** Summary of outcomes for the effect of Terlipressin in septic shock patients

| Outcomes | No. of Patients  (Studies) | Relative Effect  (95% CI) | Estimated Absolute Effects | | Quality of the Evidence (GRADE) |
| --- | --- | --- | --- | --- | --- |
|  |  |  | Assumed Risk or  Value with Control | Risk Difference or Reduction  in Value with Intervention |  |
| Mortality | 942 (10) | RR = 0.95 [0.85, 1.05] | - | 26 fewer per 1,000 (from 77 fewer to 26) | ⊕⊕⊕⊕  High |
| Duration of MV | 674 (4) | - | Mean duration ranged from  4.8 to 9.7 d | Mean duration was 1.11 d lower (2 to 0.22 lower) | ⊕⊕◯◯  Low^a^ |
| Hospital LOS | 173 (3) | - | Mean duration ranged from  10 to 21 d | Mean duration was 1.28 d lower (1.69 lower to 4.25 higher) | ⊕⊕◯◯  Low^b^ |
| ICU LOS | 866 (7) | - | Mean duration ranged from  6.4 to 23 d | Mean duration was 0.89 d lower (2.14 lower to 0.36 higher) | ⊕⊕◯◯  Low^c^ |
| AEs | 730 (5) | RR = 0.87 [0.42, 1.77] | - | 38 fewer per 1,000 (from 170 fewer to 226) | ⊕⊕⊕⊕  High |
| Digital ischaemia | 668 (3) | 1.49 [0.80, 2.77] | - | 55 more per 1,000 (from 23 fewer to 200 more) | ⊕⊕⊕◯  Moderate^d^ |
| Arrhythmia | 610(2) | 0.74 [0.32, 1.72] | - | 10 fewer per 1,000 (from 27 fewer to 29 more) | ⊕⊕⊕◯  Moderate^e^ |

GRADE = Grading of Recommendations Assessment, Development and Evaluation; MV = mechanical ventilation; RR = relative risk; MD = mean difference.

Moderate: Further research is likely to have an important impact on our confidence in the estimate of effect and may change the estimate.

Low: Further research is very likely to have an important impact on our confidence in the estimate of effect and is likely to change the estimate.

AEs: Advent events; LOS: Length of stay

^a^Low due to imprecision (median duration of mechanical ventilation reported in some studies was converted IQR/range to SD using estimation formulas) and inconsistency (inconsistent results reported across included studies with *I*^2^=49%).

^b^Low due to imprecision (median length of stay in hospital reported in some studies was converted IQR/range to SD using estimation formulas) and inconsistency (inconsistent results reported across included studies with *I*^2^=43%).

^c^Low due to imprecision (median length of stay in ICU reported in some studies was converted IQR/range to SD using estimation formulas) and inconsistency (inconsistent results reported across included studies with *I*^2^=61%).

^d^Low due to inconsistency (only three studies reporting inconsistent results).

^e^Low due to imprecision (low number of total events).
